# Nitrogen Availability and Use Efficiency in Wheat Crop as Influenced by the Organic-Input Quality Under Major Integrated Nutrient Management Systems

**DOI:** 10.3389/fpls.2021.634448

**Published:** 2021-05-19

**Authors:** Ajay K. Bhardwaj, Deepika Rajwar, Rajender K. Yadav, Suresh K. Chaudhari, Dinesh K. Sharma

**Affiliations:** ^1^ICAR-Central Soil Salinity Research Institute, Karnal, India; ^2^Indian Council of Agricultural Research, Krishi Anusandhan Bhavan-II, New Delhi, India; ^3^Regional Research Station, ICAR-Central Soil Salinity Research Institute, Lucknow, India

**Keywords:** nitrogen mineralization, nutrient release, crop residue, legume, wheat, rice

## Abstract

**Purpose:**

One of the serious constraints for the integration of organics in soil fertility plans is the release and availability of nitrogen (N) to match the critical growth stages of a crop. The interplay between organic amendment characteristics and soil moisture conditions can significantly affect the nutrient release and availability, especially for dryland crops like wheat. In this study, the effects of integrated nutrient management strategies using diverse qualities of organic amendments on daily N mineralization and its availability to plants during the full growing season of the wheat crop were analyzed in a 10-year experiment.

**Methods:**

The management included (1) F, inorganic fertilizers at 100% rate, compared to a reduced rate of inorganic fertilizers (55% N) supplemented with organic inputs via (2) GM, green manuring, (3) LE, legume cropping and its biomass recycling, (4) WS, wheat stubble retention, (5) RS, rice stubble retention, and (6) FYM, farmyard manure application, during the preceding rice season. Ion exchange resin (IER) membrane strips were used as plant root simulators to determine daily NH_4_^+^-N and NO_3_^–^-N availability in soil solution during the full wheat growing period.

**Results:**

Total available N for the full season was in the following order: GM (962 μg cm^–2^) > F (878 μg cm^–2^) > LE (872 μg cm^–2^) > FYM (865 μg cm^–2^) > RS (687 μg cm^–2^) > WS (649 μg cm^–2^). No significant differences were observed in NH_4_^+^-N availability throughout the cropping period as compared to NO_3_^–^-N which showed significant differences among management at critical crop growth stages.

**Conclusion:**

Legume biomass incorporation (GM, LE) and farmyard manure (FYM) based management provided the most consistent supply equivalent to or even exceeding 100% inorganic fertilizers at several critical stages of growth, especially at tillering and stem elongation. Integration of organics in management increased nitrogen use efficiency 1.3–2.0 times, with cereal crop residue-based management having the highest efficiency followed by legume biomass incorporation.

## Introduction

Nitrogen (N) is one of the primary nutrients for plant growth and the most used in terms of quantity of use. Decidedly, its deficiency results in indirect economic losses in crop production, while excessive use leads to nitrate loading of surface and groundwater. The latter aspect has been so widespread, the world over, for the last several decades that N management through the use of organics is mandated to be an integral part of nutrient management for crop production. Soil N availability directly affects the net primary productivity of plants through modification of nitrogen use efficiency (Aerts and Decaluwe, 1994; Cole et al., 2008). The key process contributing to N availability in soil solution is N mineralization (Ross et al., 2004; Zhou et al., 2009). Nitrogen mineralization is a biological process where soil organic N is finally transformed into NH_4_^+^ and NO_3_^–^ forms through a process-based microbial action. However, the transformation and release of N in these plant-available forms depend largely on the composition of organic matter (Manojlović et al., 2010; Marzi et al., 2020; Yamuangmorn et al., 2020). High N containing organic matter with low C/N ratio releases more N (Cordovil et al., 2005) as compared to lower N content (<1.7–2%), and high C/N ratio organic matter leading to N immobilization (Manojlović et al., 2010). Since organic sources of nutrients are important constituents of integrated nutrient management, understanding of their chemical composition, decomposition, and the rate of N mineralization is of utmost importance (Vityakon and Dangthaisong, 2005; Srinivas et al., 2006; Bhardwaj et al., 2019a).

Nitrogen content, C/N ratio, lignin, and polyphenol content of crop residues are the main controlling factors affecting net N mineralization (Oglesby and Fownes, 1992; Trinsoutrot et al., 2000; Vityakon and Dangthaisong, 2005). Organic materials, such as wheat straw with a high C/N ratio (79:1), decreased N mineralization in the early stages of decomposition due to its immobilization (Srinivas et al., 2006). This is due to the high N requirement of microbial populations involved in wheat straw decomposition (Tripathi and Mishra, 2001). Although N contained in crop residue gradually becomes available after decomposition, the addition of urea results in lowering the C/N ratio of wheat straw and consequent faster N mineralization, having no adverse effect on crop yields (Reddy et al., 2008). The Narrow C/N ratio of green manure is the main factor that favors its easy decomposition. Srinivas et al. (2006) reported that N mineralization from green manures after 3 months ranged from 71 to 82%. Similar studies have also reported by other researchers (Singh and Kumar, 1996). Singh et al. (2003) observed much higher N mineralization potential in green manure (*Sesbania aculeata*) than wheat straw. In the case of farmyard manure (FYM), although it contains inorganic N, most of the plant-available N is present in organic form and needs to be mineralized to be available for plant uptake (Sistani et al., 2008; Ling-ling and Shu-tian, 2014).

**FIGURE 1:**
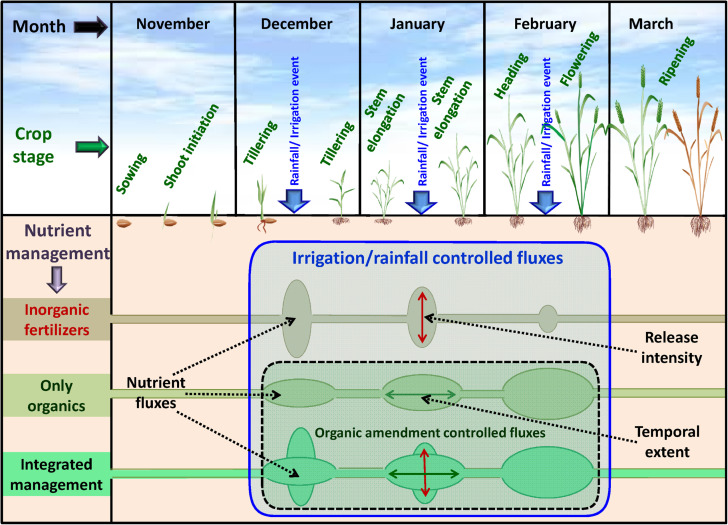
General nitrogen (N) availability scenarios under organic, inorganic, and integrated fertilization during wheat (*Triticum aestivum*) growing period.

Nitrogen mineralization from added organic matter is also influenced by abiotic factors, such as soil temperature, pH, and moisture content of the soil. The conditions during wheat cropping season are generally aerobic. Nitrogen mineralization in aerated soils is noticeably different from submerged soils. In flooded and anaerobic conditions, a higher concentration of total organic nitrogen results in higher N mineralization (Bronson et al., 2001; Bhardwaj et al., 2020). However, under aerobic conditions, soil water fluctuations, as in wheat cropping season, usually result in a greater production of NO_3_^–^-N (Nunan et al., 2000). Conversion of NH_4_^+^-N into NO_3_^–^-N takes place in the presence of O_2_ which is present in the soil. A lag has been noticed between gross N release and net N mineralization under aerobic conditions, depending on the C/N ratio of organic materials (Gale and Gilmour, 1988). Soil moisture is an important parameter affecting N mineralization under aerobic soil conditions (Kladivko and Keeney, 1987; Wang et al., 2006). Soil moisture content affects microbial activity which in turn determines mineralization rate (Figure 1). Moreover, the efficiency of microorganisms is also correlated to the C/N ratio of crop residues (Nicolardot et al., 2001). Therefore, the impact of soil moisture on N mineralization must be taken into account for a better understanding of these mechanisms in aerobic soil.

Studies on nitrogen dynamics are important to reduce the fertilizer input, minimize losses of N to the environment, and to boost residue management in the low-input agricultural systems for high crop production (Bruun et al., 2006; Jahan and Amiri, 2018). Gross N mineralization has been a center of interest for quite some time in the past (Ledgard et al., 1998; Watson and Mills, 1998). Gross rates show total N mineralization and the concurrently immobilized N by soil microbes. Therefore, it also indicates good microbial activity in the soil. The net N mineralization indicates the actual N available for plants for uptake. It has been difficult to measure net mineralization in actual field conditions due to lack of effective tools, until recently when ion exchange resin techniques came up for use (Qian and Schoenau, 2005; McSwiney et al., 2010). Most mineralization studies have been limited to laboratory experiments with a small volume of soil, and disturbed mechanical and hydraulic properties, which may not represent actual field conditions. Wheat is a staple food for the almost entire world. Net N mineralization studies during wheat growth, especially at a daily time scale, have rarely been reported. In the present study, we aimed to study net N mineralization and N availability in soil at different stages under six major integrated nutrient management systems followed for rice-wheat systems in the Indo-Gangetic region. Here we report the net N mineralization dynamics for the wheat crop grown under aerobic conditions after transplanted rice crop under anaerobic conditions.

## Materials and Methods

### Site Description

The study was done in a 10-year experiment initiated in 2005 on a soil with sandy loam texture at ICAR-Central Soil Salinity Research Institute (CSSRI), Karnal, India, located at 29.43° N and 76.58° E. This soil was one time reclaimed from alkalinity/sodicity using gypsum (CaSO_4_.2H_2_O), 10 years before initiation of experiment (in 1994), and was put under rice-wheat cropping system immediately after. At that time, immediately after reclamation, the soil (0–15 cm) recorded a pH of 8.7, cation exchange capacity = 9.5, and organic carbon = 3.2 g kg^–1^ (Yaduvanshi et al., 2013). The experimental area lies in a semi-arid subtropical climate zone with very hot summers and cool winters. The mean annual precipitation is 750 mm.

### Soil Sampling and Analysis

Soil samples were collected for 0–15 cm depth during 2015 and 2016 after the wheat crop harvesting in mid-April. For each of the four replicates, two samples were drawn at random spots within a plot and then mixed. Composite samples (4 samples for each treatment) were air dried, ground to pan through 2.0 mm sieve, and used to determine soil properties as presented in Table 1. For bulk density, samples were drawn, at the time of wheat harvest, with the help of cylindrical core samples (5 cm height × 5 cm diameter). Bulk density was determined following the methodology of Blake and Hartge (1986). The total nitrogen was determined using the Kjeldahl distillation apparatus (Pelican Equipments, Chennai, India) using methodology given by Tomov et al. (2009). Phosphorous was determined colorimetrically, and potassium was determined photometrically (Tomov et al., 2009). Soil organic carbon was determined during 2015 only after the rice season using the modified Walkley and Black’s rapid titration methodology as described by Bhardwaj et al. (2019a). The electrical conductivity (EC) and soil pH was determined using 1:2 (Soil: Water) suspension with a digital multimeter (Eutech Instruments, Singapore mode PC510) which is able to determine pH and EC simultaneously, in full range.

**TABLE 1:** Physical and chemical properties of the soils under different treatments after 10 years of management (2015).

**Treatment**	**EC (dS m^–1^)**	**pH (1:2)**	**Organic carbon (g kg^–1^)**	**Available nitrogen (kg ha^–1^)**	**Available phosphorus (kg ha^–1^)**	**Available potassium (kg ha^–1^)**	**Bulk density (g cm^–3^)**
O	0.16 *a*	8.13 *a*	4.41 *e*	115 *d*	7.6 *d*	157.9 *d*	1.69 *a*
F	0.16 *a*	7.96 *b*	5.51 *d*	162 *c*	21.5 *a*	298.6 *a*	1.55 *abc*
LE	0.15 *a*	7.93 *b*	5.78 *c*	175 *b*	17.3 *bc*	275.3 *bc*	1.43 *c*
GM	0.17 *a*	7.95 *b*	6.58 *a*	184 *a*	18.8 *b*	280.7 *b*	1.47 *bc*
FYM	0.16 *a*	8.03 *b*	6.75 *a*	171 *b*	18.2 *b*	281.9 *b*	1.64 *ab*
WS	0.15 *a*	7.98 *b*	6.25 *b*	163 *c*	16.5 *c*	261.2 *c*	1.57 *abc*
RS	0.16 *a*	7.98 *b*	5.97 *c*	161 *c*	16.1 *c*	255.4 *c*	1.54 *abc*

*For a column (parameter), treatments with same digits are not significantly different at p ≤ 0.05.*

### Experimental Layout and Treatments

The treatments included combinations of different organic amendments and inorganic fertilizers to provide for nutrient requirements of the rice-wheat cropping sequence (Table 2). With the treatments imposed for 10 years, the present investigations for N availability in soil solution using ion-exchange resin (IER) strips were carried out in 2014–15 and 2015–16 over full wheat-growing seasons (full procedure covered in later sections). The nitrogen contents of different organic amendments used under different management are provided in Table 3. There were four replications of treatments laid out in a randomized complete block design (RCBD). The annual cropping system which was followed consisted of rice (*Oryza sativa* L.) in the summer followed by wheat (*Triticum aestivum*) in the winter season. The organic amendments were added before rice transplanting and, therefore, represented residual carried over effects in wheat crop. The wheat crop was sown in the second week of November and harvested at the end of March. The management schedule and treatment details are as follows:

**TABLE 2:** Fertilizer nutrient application and organic inputs under different nutrient management systems.

**Nutrient management**	**Crop cycle**			**Fertilizer/organic inputs**			
				**Rice (at transplanting)**		**Wheat (at sowing)**	
	**May to June**	**July to Oct**	**Nov to Apr**	**Inorganic Fertilizer (N: P: K: Zn) kg ha**^–^**^1^**	**Organic**	**Inorganic Fertilizer (N: P: K: Zn) kg ha**^–^**^1^**	**Organic**
O	Fallow	Rice	Wheat	–	–	–	–
F	Fallow	Rice	Wheat	180:26:42:7	–	180:26:42:7	–
LE	Opportunity legume crop *(Vigna radiata)*	Rice	Wheat	100:16:28:0	Legume crop biomass	100:16:28:0	–
GM	Green manure crop *(Sesbania aculeata)*	Rice	Wheat	100:16:28:0	Green manure biomass	100:16:28:0	–
FYM	Fallow	Rice	Wheat	100:16:28:0	Farmyard manure (FYM)	100:16:28:0	–
WS	Fallow	Rice	Wheat	100:16:28:0	Wheat stubble	100:16:28:0	–
RS	Fallow	Rice	Wheat	100:16:28	–	100:16:28:0	Rice stubble

**TABLE 3:** Total dry matter and total nitrogen (N) inputs through organic sources used under different management during the preceding rice season.

**Amendment**	**N concentration**	**C concentration**	**C/N ratio**	**Total N input**
	**(%)**	**(%)**		**(kg ha^–1^)**
Grain Legume (*Vigna radiata*) biomass	1.71 ± 0.03	47.78 ± 1.07	27.95 ± 0.77	88
Green manure legume (*Sesbania esculeata*) biomass	2.65 ± 0.50	42.32 ± 1.13	15.99 ± 0.32	142
Farmyard manure	0.56 ± 0.06	31.36 ± 0.92	56.53 ± 4.36	21
Wheat stubble	0.27 ± 0.04	41.90 ± 0.55	157.78 ± 7.77	11
Rice stubble	0.46 ± 0.07	42.42 ± 0.33	93.1 ± 3.48	18

1. F: Wheat (November–April) was grown with 100% inorganic fertilizer (N, P, K, and Zn) input after rice (July–October) with 100% inorganic fertilizer inputs only. The fertilizer application was done in three splits at time = 0, 21, and 42 days after sowing. No organic inputs were given.
2. LE: Legume crop, *Vigna radiata*, was grown in the summer lean period (April–July) between wheat and rice as an “opportunity crop.” The Vigna seeds were sown in the first week of April, immediately after wheat harvest. In the first week of July (after ∼ 90 days of sowing) pods were harvested and the remaining plant biomass was incorporated into the soil using a power tiller just before rice transplanting in July. Wheat was sown in November with reduced (55% N, P, and K) inorganic fertilizer inputs.
3. GM: A green manure crop, *Sesbania aculeata*, was grown in the lean period (May–July) between wheat and rice. The green manure crop was sown on or around the 20th of May every year after wheat harvest. After 35–40 days of sowing, the green manure crop was incorporated into the soil using a power tiller, just before rice transplanting in July. Wheat was sown in November with reduced (55% N, P, K) inorganic fertilizer inputs.
4. FYM: Farmyard manure (FYM) at the rate of 10 t ha^–1^ was incorporated in the soil just before soil puddling and transplanting of rice in July. Wheat was sown in November with reduced (55% N, P, K) inorganic fertilizer inputs.
5. WS: 30 cm standing stubble (∼1/3 of the total straw) of wheat was retained at the time of harvesting of wheat. It was dry plowed into the soil before soil puddling and transplanting of rice in July. Rice was transplanted in July and wheat was sown in November both with reduced (55% N, P, and K) inorganic fertilizer inputs.
6. RS: 30 cm standing stubble (∼1/3 of the total straw) of rice was retained at the time of harvesting of rice. It was dry plowed into the soil at the time of wheat sowing. Wheat was sown in November with reduced (55%) fertilizer inputs. Rice was transplanted in the following season (July–October) with reduced (55% N, P, K) inorganic fertilizer inputs only.
7. O (Absolute control): No fertilizers (inorganic and organic) were applied.

The absolute control, with no fertilizer nutrient application, was not considered for mineralization studies, except for calculating nitrogen use efficiency, assuming that no-fertilizer application (soil mining) is an unsustainable option. The inorganic N fertilizer application was in three equally split doses for all management using urea and DAP (Dia-Ammonium Phosphate) granules. The first 1/3 dose was immediately before sowing of wheat (DAP and urea), the second 1/3 dose was at 21 days after sowing (DAS) using urea, and the third 1/3 dose was at 42 DAS using urea. Irrigations @ 10 cm single event were applied at monthly intervals constituting around 3–4 irrigations during the full growing season from the first week of November to mid-April. During the study period, 3 irrigations per season were applied at 30, 60, and 90 DAS (exact data mentioned in results).

### Nitrogen Availability in Soil Solution

Nitrogen availability in soils was determined throughout the growing season using the ion exchange resin (IER) membranes. Membrane strips (cation and anion separately) of 2.5 cm by 10 cm size were cut out from large commercially available sheets (General Electricals, Watertown, MA, United States). The strips were charged by dipping and shaking in 0.5 mol/L HCl for 1.2 h, and then in 0.5 mol/L NaHCO_3_ for 5 h. Finally, they were rinsed with deionized water. The resin strips were placed in vertical slots made in the treated soils, and each slot was closed firmly so that strips become contacted with soil. Both cation and anion strips were placed 5 cm apart, were kept in the soil for 15 days interval, and were replaced with new ones immediately after removing the previous set of strips. This process was continued for the entire cropping season. After removal from the soil, the strips were rinsed with deionized water to remove any adhering soil. For a treatment, both cation and anion strips were stored and transported together in a vial for extracting NH_4_^+^ and NO_3_^–^ in the laboratory. For extraction, 70 ml of 2 molL^–1^ KCl was added to the vials with strips, shaken for 1 h, and decanted into a scintillation vial. The extracts were analyzed using Kjeltec 2200 (Foss, Hillerod, Denmark) for NH_4_^+^-N and NO_3_^–^-N.

### Nitrogen Use Efficiency

The nitrogen use efficiency (NUE) was calculated as:

$$\text{NUE}=\frac{\left(Y-Y_{0}\right)}{F}$$

where Y is the yield in the treatment plots, Y_0_ is the yield in the control (no fertilizer application), and F is the amount of nitrogen applied, all in kg ha^–1^. Nitrogen use efficiency was calculated based on the N applied as inorganic fertilizers in each management, as well as based on the total N applied through inorganic fertilizers plus organic inputs.

### Amendment Sampling and Analyses

Biomass samples were drawn for all amendments and analyzed for total N. Samples for legume crop (*Vigna radiata*) and green manure crop (*Sesbania aculeata*) were taken in June, at the time of harvesting and incorporation into the soil. FYM samples were taken at time of their application to the treated soil. Rice stubble was sampled at the rice harvesting (October-end) and wheat stubble was sampled at the time of wheat harvesting (mid-April), using 1 m^2^ quadrat. Table 3 provides the information about the C and N concentrations of organic inputs in each type of management. The samples of organic materials were drawn during 2015 and 2016 for all analysis.

### Statistical Analysis

All the data including growth parameters were statistically analyzed using SAS. For N availability data at the different growth stages, separation of means was subjected to Tukey’s honestly significant difference test using JMP 9.0 (SAS Institute Inc., Cary, NC, United States). The graphing was done using Origin v.8.5 software (Originlab Corporation, Northampton, United States). Correlation analysis was conducted to identify relationships between the measured parameters. All tests were performed at the 0.05 significance level.

## Results

**FIGURE 2:**
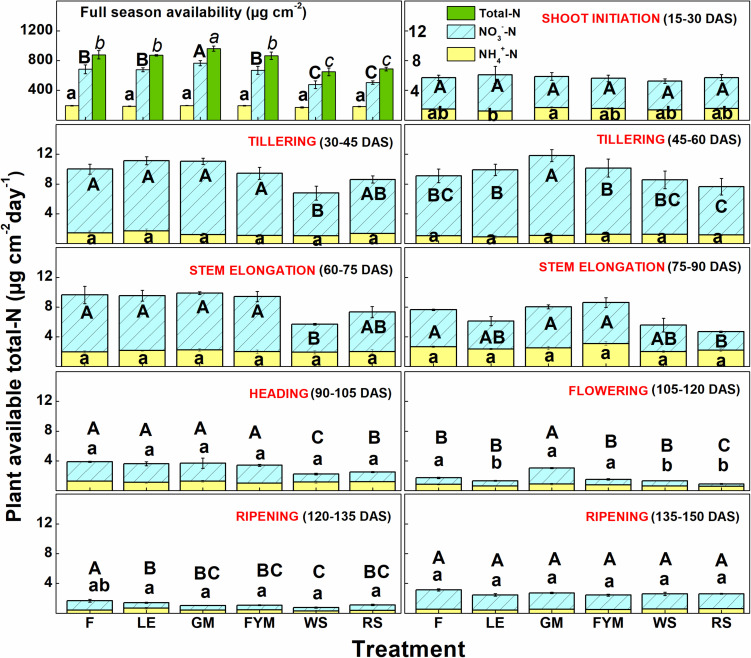
Full season and daily NO_3_^–^-N, NH_4_^+^-N, and total N availability scenarios with days after sowing (DAS) for wheat (*Triticum aestivum*) crop (stage-wise averaged for 2014–15 and 2015–16). For respective parameter, treatments with same digits are not significantly different at *p* ≤ 0.05, using Tukey’s HSD. IER, Ion exchange resin strips. Small letters represent differences for NO_3_^–^-N, small italic letters represent differences for NH_4_^+^-N, and capital letters represent differences for total plant-available N. Error bars denote ± 1SD. Management: *F* = 100% inorganic fertilizers, LE, Legume crop (*Vigna radiata*) in rotation and its biomass incorporation + 55% inorganic fertilizers, GM, Green manuring with *Sesbania aculeata* + 55% inorganic fertilizers, FYM, farmyard manure incorporation + 55% inorganic fertilizers, WS = 1/3rd wheat stubble retention and incorporation + 55% inorganic fertilizers, RS = 1/3rd rice stubble retention and incorporation + 55% inorganic fertilizers.

**FIGURE 3:**
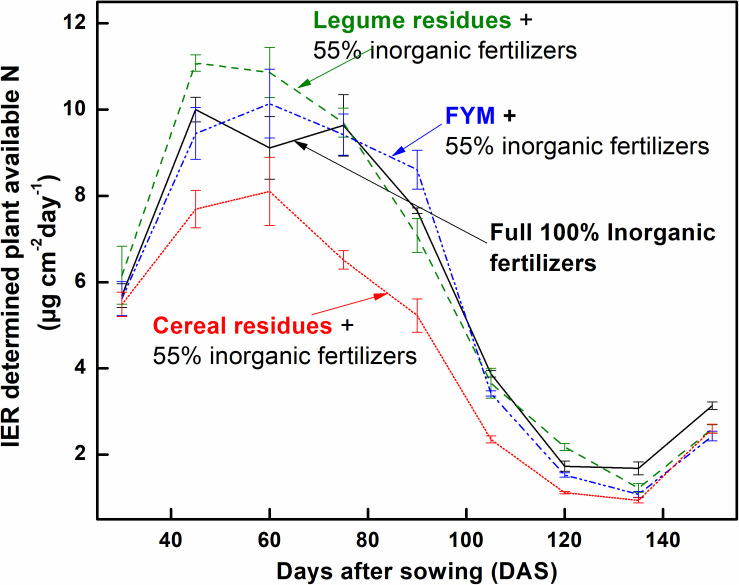
Trends in IER determined plant-available total-N in soil solution during the wheat (*Triticum aestivum*) crop growth under different management. Error bars denote ± 1SD. IER, Ion exchange resin strips. Treatments are described in Figure 2 legend. The curve for crop residues denotes averaged values for WS and RS treatments, and the curve for legumes denotes averaged values for GM and LE treatments.

**FIGURE 4:**
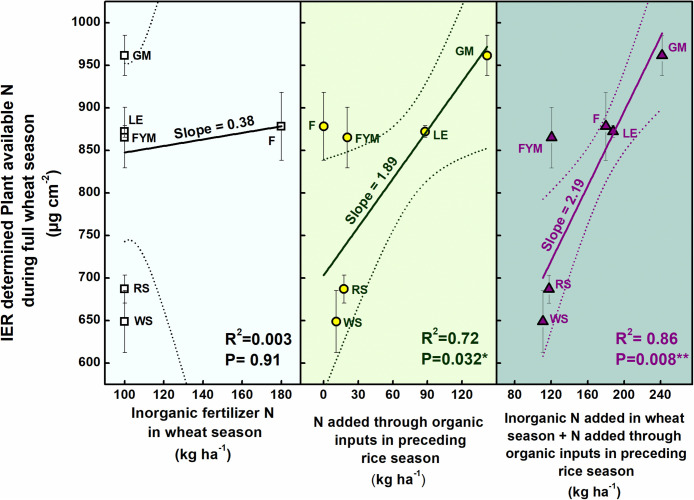
Relations between nitrogen (N) applied through organic and inorganic sources and IER (ion exchange resin) determined N availability during wheat (*Triticum aestivum*) growing season. Error bars denote ± 1SD. Treatments are described in Figure 2 legend.

**FIGURE 5:**
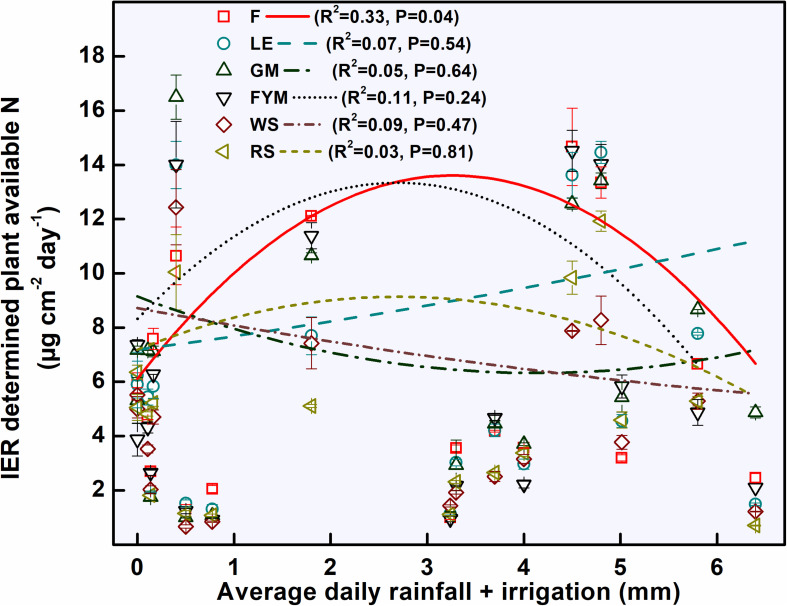
Relation between daily water availability (rainfall + irrigation) and IER (ion exchange resin) determined plant-available nitrogen (N) during the wheat growing season. Error bars denote ± 1SD. Treatments are described in Figure 2 legend.

**FIGURE 6:**
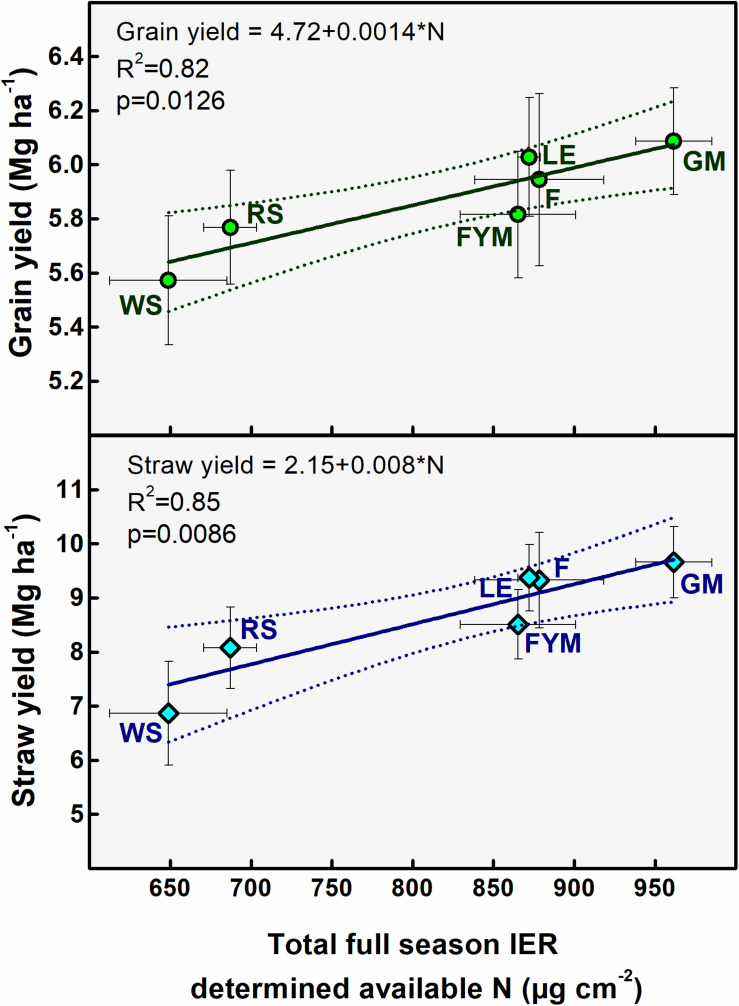
Relation between full-season IER (ion exchange resin) determined plant-available nitrogen (N) and averaged grain and straw yields from 2011 to 2018. Vertical error bars denote ± 1SD and horizontal bars denote ± 1SD. Treatments are described in Figure 2 legend.

**FIGURE 7:**
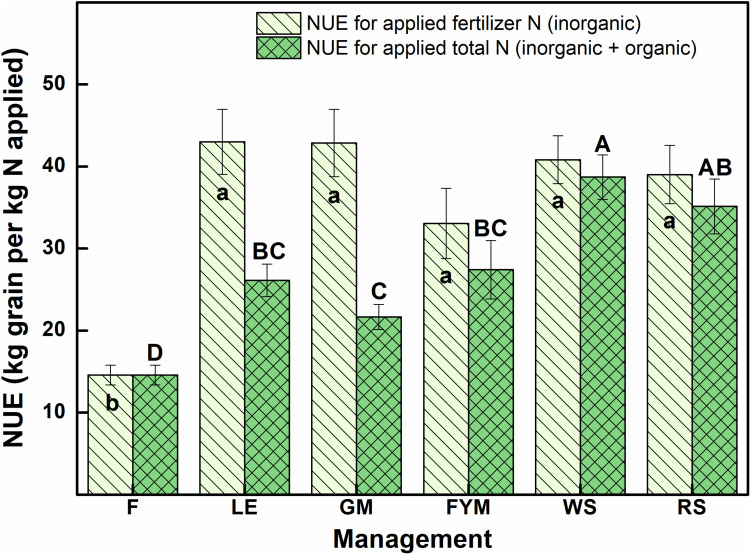
Nitrogen use efficiency (NUE) of the wheat crop in response to different management after 10 years of management (averages for 2014–15 and 2015–16). Error bars denote ± 1SD. For respective parameters, treatments with the same digits are not significantly different at *p* ≤ 0.05, using Tukey’s HSD. Small letters represent differences for applied fertilizer N (inorganic), and capital letters represent differences for total applied N (inorganic + organic), small italic letters represent differences for NH_4_^+^-N and capital letters represent differences for total plant-available N. Treatments are described in Figure 2 legend.

Ion exchange resin (IER) strip sorbed nitrogen (N) represented plant-available amounts, and it showed significant differences at different stages of wheat crop growth with different management strategies (Figure 2). During the full season, total-N availability was maximum in GM (962 μg cm^–2^) followed by F (878 μg cm^–2^), LE (872 μg cm^–2^), and FYM (865 μg cm^–2^) whereas in RS and WS the availability of total-N was 29 and 33% lower, respectively, compared to GM. Overall, NO_3_^–^-N was the dominant form of available nitrogen than NH_4_^+^-N. Available NO_3_^–^-N was around four times that of NH_4_^+^-N in F, LE, GM management, and around three times that in FYM, WS, and RS managements, for full season. The NH_4_^+^-N was available in much lower amounts, and there were no significant differences in its availability amongst different management, at any growth stage, except at shoot initiation where it was maximum in GM. On the other hand, NO_3_^–^-N availability throughout the cropping period was highly variable, with significant differences among management. Initially, up to 30 DAS, NO_3_^–^-N availability, on average, in managements with organics integrated and reduced rates (55%) of fertilizers was found comparable to F (100% inorganic fertilizer) (Figure 2). From 30 to 75 DAS, total N availability increased twofold as compared to 15–30 DAS and then gradually decreased sevenfold during the subsequent stages (75–150 DAS). Among the management, legumes (LE, GM) surpassed other treatments, especially F and WS, in terms of NO_3_^–^-N availability in soil solution at 15–30 DAS. The availability from LE peaked (11.12 ± 0.56) at early tillering (30–45 DAS) and hence after declined. In case of GM the availability (conversely release from organic matter) peaked (11.81 ± 0.64) at 45–60 DAS, coinciding with late tillering stage. Also, at the tillering stage, GM had significantly more availability of NO_3_^–^-N than any other treatment, and it was 40 and 25% more than RS and F managements. Cereal crop residue-based management (WS, RS) as compared to legumes and farmyard manure had significantly lower N availability at all stages except first 30 days (where basal and first split fertilizer is also added and ripening stage). From 135 to 150 DAS, a period coinciding with the maturity of crop and very dry-soil conditions, no significant differences were observed among all managements in terms of N availability. The total N availability declined to below 4 μg cm^–2^ day in all treatments beyond heading stage (90 DAS). The 100% inorganic fertilizer treatment also availed significantly comparable total N at stages where irrigation was given. Irrigation were given at 31, 64, and 95 DAS during 2014–15 and at 33, 60, and 91 DAS during 2015–16, coinciding with early tillering, stem elongation, and heading stage. For dry season crops like wheat, rainfall/irrigation events instigate release and dissolution of nutrients especially nitrogen (Figure 1). The intensity of release is controlled by the fertilizer characteristics. Quick release and dissolution of the nutrient is provided by the inorganic fertilizer for plant uptake as well as loss by leaching. On the other hand organic amendment release is slow (water aided decomposition) yet the release is consistent for long period and nitrogen has less chances of loss (Figure 2). Daily availability trends of total-N among management revealed that the organics with high N availability and low C: N ratio (<50) (legumes, farmyard manure, and fertilizer alone) have better N mineralization and availability than those with high C:N ratio (cereal crop residue based) (Figure 3). The trends indicate that reduction in organic fertilizer N may not suffice for wheat crop growth under integrated management with cereal (rice, wheat) crop residue. The plant-available N in soil solution related best to the combined effects of inorganic N applied in the wheat season plus the organic N added via the organic amendments which were added in the preceding season of rice (Figure 4). Average daily rainfall + irrigation during a period of sampling (IER strip residence period in soil) were related to plant N availability. Total-N availability in soil solution significantly correlated to daily rainfall+irrigation in the case of 100% inorganic fertilizers (Figure 5). With increased daily water application (rainfall+ irrigation), F (and FYM to a lesser extent) showed an increase in N release into soil solution up to 3 mm daily water application, and thereafter decreased with an increase in water application levels. However, in the case of GM, LE, WS, and RS no significant relations could be found. The relation provided indirect evidence regarding water application optimization for enhancing fertilizer use efficiency in dryland crops like wheat. The relationship of total available N during the full wheat growing season with grain and straw yield indicated significant relation between available N and grain yield (*P* = 0.01) as well as available N and straw yield (*P* = 0.008; Figure 6). In terms of nitrogen use efficiency (NUE), the cereal crop residue incorporation (WS, RS) had the maximum efficiency followed by legume biomass incorporation (GM, LE) and FYM, and the least efficiency was obtained in the 100% inorganic fertilizer application (F) (Figure 7). When only inorganic fertilizer N was considered, all integrated management improved efficiency, equally, compared to F. On basis of the total N applied, the NUE decreased in the order: cereal crop residues (WS, RS) > legume crop residues (GM, LE) > FYM > only inorganic fertilizers (F).

## Discussion

Overall, nitrogen (N) availability status for wheat crop indicated comparable full season N availability in F (100% inorganic fertilizer application), LE (legume opportunity crop *Vigna radiata* and its biomass incorporation), GM (green manuring with *Sesbania aculeata*), and FYM (farmyard manure incorporation @ 10 Mg ha^–1^). Crop residue-based management, RS (rice stubble incorporation), and WS (wheat stubble incorporation) showed significantly lower N availability. Except for the first 60 days after sowing (DAS) when three split inorganic fertilizer applications were done (100% fertilizer split into three doses for F, and 50% fertilizer split into 3 doses for other management), the availability was significantly similar but cereal crop residue-based management lagged 60 DAS onwards in terms of plant-available N in soil solution.

The N availability scenarios in wheat crop were best represented by the combined effects of inorganic fertilizer application as well as residual N from organic amendments added in the rice season (Bhardwaj et al., 2020). Only combined effects could explain well the availability scenarios (Figure 4). In the case of wheat crop with relatively drier/near-field-capacity soil condition, F management matched legume-based (GM, LE) and manure-based (FYM) managements in terms of N availability. This meant that in the case of legumes (GM, LE) and farmyard manure (FYM), 50% cut down of inorganic fertilizer is compensated by the residual N from the organic amendment application in the preceding rice season, and secondly, the inorganic fertilizer losses were lower under drier conditions and therefore effects of inorganic fertilizer lasted longer than under flooded conditions as prevailed in the case of rice in the preceding season (Bhardwaj et al., 2020).

The characteristics of the organic amendment also played a role as evident from the case of FYM and cereal crop residue (WS, RS) based management. In the case of FYM, instead of lower total applied N (inorganic + organic), N availability matched F and LE. In FYM, the applied N was in the range of RS and WS. Therefore, the mixed effects of total N, C/N ratio of amendments, and decomposability played a decisive role. Perhaps, ready availability played the most significant role under such conditions as prevailed in wheat season (drier and near field capacity conditions with intermittent low-intensity rain events or irrigations) (Figure 1). Under drier crop conditions, the quality of organic matter inputs plays a significant role. Low-quality organic inputs (low C/N ratio, high lignin, etc.) may increase organic carbon in soil but not productivity (Goyal et al., 1992; Snapp et al., 1998). In the case of WS (wheat stubble incorporation) and RS (rice stubble incorporation), N availability was much lower than other managements. These are the amendments with a wider C/N ratio as well as a lower total N compared to legumes. Cereal crop residues do not meet the critical N content of 1.8–2.0% needed by microbes and may immobilize N (Snapp et al., 1998). Though the immobilization is temporary and can be offset by integrating inorganic fertilizers (Murwira and Kirchmann, 1993), this does not seem to hold good enough for dry season crops like wheat. The N availability in soil solution corresponded to total N added in the management (Figure 4), but C/N ratio and decomposability (related to N release) played a significant role as could be noticed in case of FYM wherein the total N application was equal to crop residues based management (RS, WS), yet the N availability was much higher than these managements. Several studies have suggested that crop residue induced N immobilization during initial stages and N release during later stages depends on several abiotic and biotic factors (Corbeels et al., 2000; Roy et al., 2011). Since the nutrient availability is driven by water, organic sources with the quick release of nutrients (at each rain/irrigation event) would show better availability to plants. Residue from legumes (green manure crop *Sesbania* and opportunity legumecrop *Vigna*) has considerably higher contents of N as well as narrower C/N ratio, thus leading to considerably more and quick release of N into soil solution for plant uptake (Schomberg et al., 1994; Bhardwaj et al., 2020). The effects have been found to persist for longer periods, even years, with annual release as much as 5–10% per year, providing benefits to subsequent crops (Fillery, 2001; Peoples et al., 2009; Khakbazan et al., 2014; St. Luce et al., 2015).

The relationship between daily water application (rainfall + irrigation) also revealed the importance of water management. While the N availability in managements with organic amendments did not relate significantly to water application, in the case of F (100% inorganic fertilizers) the availability peaked at 3 mm (*P* = 0.05) and thereafter decreased with an increase in the water application. On the whole, NO_3_^–^-N was the dominant form in the soil in the wheat season with almost 70–80% of N availability in NO_3_^–^-N form and 20–30% in NH_4_^+^-N form. The ultimate fate of NH_4_^+^-N produced in aerobic agricultural soils is NO_3_^–^-N where NH_4_^+^-N is very low and has short residence time, while under anaerobic conditions major availability is in form of NH_4_^+^-N since nitrification is inhibited (Robertson, 1997; Murphy et al., 1998; Bhardwaj et al., 2019b). Moisture availability controls the mobility of nutrients near (to the plants) and far (losses via leaching) as well as by controlling the microbial and enzymatic activity affecting mineralization (Bhardwaj et al., 2020). Therefore the optimum moisture content reflects the best balance between the availability to plants and losses beyond the reach of plant roots.

The nutrient use in a system has the primary goal of providing optimum nutrition to the crop plants with minimum losses. The use efficiency of a nutrient takes care of some but not all aspects of nutrient use in crop production. The cereal crop residue-based management lagged in total N availability to crop plants compared to legume residue-based management but the former’s use efficiency was significantly higher than the latter. In all, the integrated nutrient management with the incorporation of cereal crop residue or green manuring or grain legume cropping and its biomass incorporation or FYM, along with 50% inorganic fertilizer application, significantly increased nitrogen use efficiency and grain yield over 100% inorganic fertilizer use. An optimum balance is struck when a practice provides both high use efficiency and availability to plants. Integration of legume crops (green manuring with legume, inclusion of grain-legume crop in rotation) provides a balance of efficiency and availability of nitrogen, the most important nutrient element for crop production as well as in terms of environmental performance.

## Conclusion

Nitrogen release characteristics of organic materials determine the effectiveness of an integrated nutrient management strategy. Wheat (*Triticum aestivum*) being a dry season crop the nitrogen availability, and its interactions with water availability, play a significant role. Strategies with quick release and availability of nitrogen should be preferable under the drier conditions which prevail during the wheat crop. For integrated nutrient management, organic amendments with a narrow C/N ratio (legume crop residues) as compared to those with a wider C/N ratio (cereal crop residues) provided the best nitrogen availability scenarios for the wheat crop. Therefore, for the wheat crop, cutting down inorganic fertilizers to almost half and supplementing with Sesbania-based green manuring or with grain legume cropping and incorporation of its biomass into the soil at harvest, or by application of FYM are the best options for integrating organics into the nutrient management schemes for wheat. These managements have either better or comparable (to 100% inorganic fertilizer application) mineralization of nitrogen at all critical stages of wheat crop. On the other hand, cereal crop residue-based managements have the best nitrogen use efficiency. In all, the integration of legumes and their biomass incorporation into soil achieved optimum balance for wheat nitrogen-nutrition. Recycling cereal crop residues in moderate quantities (1/3rd) by incorporation into soil, along with reduced rates of inorganic fertilizers, also provided comparable benefits as compared to 100% inorganic fertilizers.

## Data Availability Statement

The data supporting the findings in the manuscript is either available within the article or in supporting information and is freely available from the corresponding author on reasonable request.

## Author Contributions

DR contributed in data collection, analysis, and manuscript writing. RY, SC and DS interpreted the data and wrote the manuscript. All authors contributed to the article and approved the submitted version.

## Conflict of Interest

The authors declare that the research was conducted in the absence of any commercial or financial relationships that could be construed as a potential conflict of interest.
